# Double-Stranded RNA Attenuates the Barrier Function of Human Pulmonary Artery Endothelial Cells

**DOI:** 10.1371/journal.pone.0063776

**Published:** 2013-06-03

**Authors:** Zoltán Bálint, Diana Zabini, Viktoria Konya, Chandran Nagaraj, Attila G. Végh, György Váró, Imola Wilhelm, Csilla Fazakas, István A. Krizbai, Akos Heinemann, Horst Olschewski, Andrea Olschewski

**Affiliations:** 1 Experimental Anesthesiology, Department of Anesthesia and Intensive Care Medicine, Medical University of Graz, Graz, Austria; 2 Ludwig Boltzmann Institute for Lung Vascular Research, Graz, Austria; 3 Division of Pulmonology, Department of Internal Medicine, Medical University of Graz, Graz, Austria; 4 Institute of Biophysics, Biological Research Centre of the Hungarian Academy of Sciences, Szeged, Hungary; 5 Institute of Experimental and Clinical Pharmacology, Medical University of Graz, Graz, Austria; University of Giessen Lung Center, Germany

## Abstract

Circulating RNA may result from excessive cell damage or acute viral infection and can interact with vascular endothelial cells. Despite the obvious clinical implications associated with the presence of circulating RNA, its pathological effects on endothelial cells and the governing molecular mechanisms are still not fully elucidated. We analyzed the effects of double stranded RNA on primary human pulmonary artery endothelial cells (hPAECs). The effect of natural and synthetic double-stranded RNA (dsRNA) on hPAECs was investigated using trans-endothelial electric resistance, molecule trafficking, calcium (Ca^2+^) homeostasis, gene expression and proliferation studies. Furthermore, the morphology and mechanical changes of the cells caused by synthetic dsRNA was followed by *in-situ* atomic force microscopy, by vascular-endothelial cadherin and F-actin staining. Our results indicated that exposure of hPAECs to synthetic dsRNA led to functional deficits. This was reflected by morphological and mechanical changes and an increase in the permeability of the endothelial monolayer. hPAECs treated with synthetic dsRNA accumulated in the G1 phase of the cell cycle. Additionally, the proliferation rate of the cells in the presence of synthetic dsRNA was significantly decreased. Furthermore, we found that natural and synthetic dsRNA modulated Ca^2+^ signaling in hPAECs by inhibiting the sarco-endoplasmic Ca^2+^-ATPase (SERCA) which is involved in the regulation of the intracellular Ca^2+^ homeostasis and thus cell growth. Even upon synthetic dsRNA stimulation silencing of SERCA3 preserved the endothelial monolayer integrity. Our data identify novel mechanisms by which dsRNA can disrupt endothelial barrier function and these may be relevant in inflammatory processes.

## Introduction

Endothelial function is essential for vascular integrity. The endothelium provides a barrier, regulates vascular tension, and is involved in angiogenesis and haemostasis. Local and systemic inflammation, however, can impair endothelial function and can lead to cellular damage increasing endothelial permeability and loss of epithelial barrier function [1], [2]. Endogenous RNA release and circulating RNA like virus–associated double stranded RNA (dsRNA) may contribute to the development of endothelial dysfunction. Endothelial cells express toll-like receptor 3 (TLR3) [3] which is activated by dsRNA [4], [5]. The activation of TLR3 affects cell homeostasis [6], [7] and causes changes at functional [8], [9], as well as inflammatory gene expression level [10]. At cellular level, dsRNA induces a signaling cascade [11], [12] leading to TLR3 receptor upregulation [4], [13]. At organ level, repeated and long-term administration of synthetic dsRNA causes inflammation [14], [15] and leads to impairment of lung function in mice [16]–[18]. However, the biological activity of circulating extracellular RNA is poorly understood.

Recently, an extracellular RNA-induced activation of VEGF has been shown, leading to increased permeability of cerebral endothelial cells, which are the main components of the blood brain barrier [19]. This hyperpermeability can occur due to exposure of the cells to total RNA [8] or the synthetic analogue of dsRNA, polyinosinic-polycytidylic acid (Poly I:C) [20] and can lead to disintegration of adherens junctions [21]. Endothelial permeability regulation [22] and function [23], [24] is a Ca^2+^-dependent process [1], [25]. A rise in intracellular Ca^2+^ in the ECs occurs through Ca^2+^ influx or by release from the sarco-endoplasmic reticulum (SER) resulting in plasma membrane-located Ca^2+^ channel activation [26], [27]. To maintain the Ca^2+^ homeostasis of the cell, the Ca^2+^ stores are refilled by the SER-membrane-located sarco/endoplasmic reticulum Ca^2+^ ATPase (SERCA) [28]. SERCA is encoded by three homologous genes: SERCA1, SERCA2 and SERCA3 [29], out of these in human pulmonary artery endothelial cells (hPAECs) only SERCA2 and SERCA3 isoforms are expressed [30], [31]. However, SERCA plays an important role not only in the Ca^2+^ homeostasis [24], [30], [32], but it is vital for cell cycle control [33], proliferation and regulation of cellular permeability as well.

In the present study we investigated alternative pathways of dsRNA on primary hPAECs. Changes in cell morphology, permeability, cellular junctions, mechanical properties and Ca^2+^ homeostasis were characterized. Furthermore, we assessed the effects of natural and synthetic dsRNA on gene expression, proliferation of hPAECs and on SERCA.

## Materials and Methods

### Cell Culture

Human pulmonary artery endothelial cells (hPAECs) were obtained from Lonza (Allendale, New Jersey) and they were cultured according to the manufacturer’s instructions. The endothelial specific media (VascuLife, Lifeline) was changed every third day. Cells in passages 5–9 were used for the experiments and the endothelial phenotype was regularly checked for von Willebrand factor expression.

### Solutions and Chemicals

Poly I:C was purchased from Amersham Pharmacia, Λ-DNA from Fermentas (SD0021), LY-294002 and 2,5-Di-*t*-butyl-1,4-benzohydroquinone (BHQ) was received from Sigma. Double-stranded RNA (sense: 5′-UAC-ACC-GUU-AGC-AGA-CAC-CdTdT-3′, antisense: 5′-GGU-GUC-UGC-UAA-CGG-UGU-AdTdT-3) was from Qiagen. Total cellular RNA was isolated from hPAECs with the RNeasy Mini Kit (Qiagen). PhosphoAKT and AKT antibodies were obtained from Cell Signalling Technology Inc. All chemicals were dissolved and diluted to the desired concentration in experimental solution containing in mM: 145 NaCl, 5.5 KCl, 1.8 CaCl_2_, 1 MgCl_2_, 10 Glucose and 10 HEPES. The pH was set to 7.4. All the solutions were freshly prepared on the day of the experiment and stored at 4°C until they were used. The experiments were carried out at room temperature unless otherwise stated.

### Transendothelial Electrical Resistance Measurement (TEER)

For measuring the cellular barrier properties hPAECs were grown until confluence on semi-permeable inserts. The TEER was measured using an Endohm chamber connected to an EVOM resistance meter (World Precision Instruments, Sarasota, USA) and TEER values were recorded every 30 min for the first 5 hours and additionally at 18, 20, 22 and 24 hour timepoints after treatment with 25 µg/mL Poly I:C, 25 µg/mL dsRNA, 2.5 µg/mL totalRNA, 2.5 µg/mL Λ-DNA or control solution. The measurements were carried out at 37°C. The resistance of a blank filter insert filled with the control media was subtracted as background value from the total resistance of each culture insert [34].

### Permeability Measurement with Fluorescently Labelled Dextran

To assess the permeability changes of the endothelial monolayer, hPAECs were grown until confluence on semi-permeable inserts and incubated for 24 h with 25 µg/mL Poly I:C, 25 µg/mL dsRNA, 2.5 µg/mL totalRNA, 2.5 µg/mL Λ-DNA, 25 µM LY-294002, 30 µM BHQ or control solution (vehicle control). After treatment, FITC-labelled dextran was added to the upper compartment of the insert (ECM640 *In Vitro* Vascular Permeability Assay Kit, Chemicon). The fluorescence of the solution from the bottom well was measured with a fluorescent plate reader (λex = 485 nm, λem = 525 nm; Optima, BMG Labtech).

### Quantitative RT-PCR

For quantitative RT-PCR and as a stimulation agent, total cellular RNA from hPAECs was isolated with the RNeasy Mini Kit from Qiagen. The protocol for purification of total RNA from cells using spin technology was followed (Cat. No./ID: 74104). Additionally, DNase digestion during RNA isolation was carried out with the RNase-Free DNase-Set from Qiagen (Cat. No./ID: 79254). The Agilent 2001 Bioanalyzer and Agilent RNA 6000 Nano Assay Protocol were used to quantify the concentration and the purity of the isolated total RNA. The total cellular RNA isolated from endothelial cells was converted to cDNA using a RevertAid H Minus First Strand cDNA Synthesis kit (Fermentas). PCR amplifications were performed by AB7900 (Applied Biosystems) using the following primers: Hs_GAPDH_2_SG (QT01192646) - *GAPDH*, Hs_ATP2A3_1_SG (QT00087220) - *SERCA3*; Hs_ATP2A2_1_SG (QT00077231) - *SERCA2*; and the SYBR Green I Master Mix (Qiagen). Amplifications were performed in triplicates and the mean threshold cycle (Ct) reading was used. Gene expressions were quantified relative to the housekeeping gene (GAPDH) and normalized to the expression level of untreated control samples (delta-delta Ct method).

### Immunofluorescent Staining

HPAECs were seeded on chamber slides and were grown until confluence then they were treated for 24 h with 25 µg/mL Poly I:C, 25 µg/mL dsRNA, 2.5 µg/mL totalRNA, 2.5 µg/mL Λ-DNA, 25 µM LY-294002, 30 µM BHQ or kept untreated (control). The cells were fixed with a buffer containing 100 mL phosphate buffered saline solution (PBS) pH 7, 650 mg Na_2_HPO_4_, 400 mg NaH_2_PO_4_, 1.5 mL Methanol, 10 mL 4% Formaldehyde. The slides were washed with pre-warmed Hepes buffered saline solution (HBSS) for 20 min at room temperature (RT). After washing the cells for 5 min in PBS, they were covered for 30 min with 100 nM Glycerol (RT). 3×5 min washing steps with PBS were performed and followed by permeabilization with 0.1% Triton-X-100 for 30 min at RT. Afterwards 3×5 min PBS washing steps were performed. The cells were blocked for 30 min with 3% BSA in PBS at RT, than they were washed again for 5 min. The primary antibody (VE-Cadherin, 1∶200 dilution, Abcam; ZO-1, 1∶100 dilution, Zymed) was added to the cells for 30 min (RT). After washing for 3×5 min, the cells were incubated at RT for 30 min with goat anti-mouse antibody conjugated with AF-594 (1∶500 dilution, Molecular Probes) in dark. Finally, the cells were counterstained with DAPI to identify the nuclear DNA. Duplicates processed without primary antibodies served as negative controls. Fluorescence was imaged using a Zeiss 200 M inverted epifluorescent microscope.

For phalloidin staining the cells were fixed with 4% formaldehyde for 30 min at 4°C, permeabilized using acetone at −20°C for 10 min. After blocking with 3% bovine serum albumin for 30 min, the coverslips were incubated with Alexa488-phalloidin (Molecular Probes). Mounting was performed in anti-fading embedding medium (Biomeda) and the distribution of the signal was imaged using a Nikon Eclipse TE2000U photomicroscope with epifluorescent capabilities connected to a digital camera (Spot RT KE).

### Western Blotting

Protein extracts were prepared from hPAECs in RIPA buffer containing Protease-Inhibitor and Phosphatase-Inhibitor tablet (Roche, Vienna, Austria). Equivalent amounts of protein were resolved on 10% SDS polyacrylamide gels and proteins were transferred to the nitrocellulose membrane. Nonspecific antibody binding was blocked by incubation in 5% (m/v) non-fat dry milk powder in TBST (20 mM Tris-Cl, pH 7.5, 150 mM NaCl, 0.1% (v/v) Tween 20) at room temperature for 1 h. Afterwards, the blots were incubated overnight with a 1∶1000 diluted primary antibody at 4°C. After washing the membranes in TBST buffer and incubating with 1∶2000 diluted horseradish-peroxidase conjugated anti-IgG secondary antibody for 1 h at room temperature, specific immunoreactive signals were detected by enhanced chemiluminescence (ECL, Amersham, Freiburg, Germany).

### Live Cell Ca^2+^ Imaging

The cells were cultured on gelatine coated, 25 mm glass cover slips. After reaching confluence, they were treated for 24 h with 25 µg/mL Poly I:C, 25 µg/mL dsRNA, 2.5 µg/mL Λ-DNA, 2.5 µg/mL total RNA or left untreated (control). The cover slips were loaded with fura-2/AM (2 µmol/L) in dark for 45 min followed by a washing step in experimental solution as previously described [35]. After 15 min, the single glass cover slip was mounted on the stage of a Zeiss 200 M inverted epifluorescence microscope coupled to a PolyChrome V monochromator (Till Photonics, Germany) light source in a sealed temperature-controlled RC-21B imaging chamber (Warner Instruments, USA) and perfused with prewarmed solution (30°C). Fluorescence images were obtained with alternate excitation at 340 and 380 nm. The emitted light was collected at 510 nm by an air-cooled Andor Ixon camera (Andor Technology, Ireland). Measurements were made every 3 s. Background fluorescence was recorded from each cover slip and subtracted before calculation. The acquired images were stored and processed offline with TillVision software (Till Photonics, Germany). [Ca^2+^]_i_ was calculated as described by Grynkiewicz *et al.* [36]. Maximal and minimal ratio values were determined at the end of each experiment by first treating the cells with 1 µmol/L ionomycin (maximal ratio) and then chelating all free Ca^2+^ with 10 mmol/L EGTA (minimal ratio). Cells that did not respond to ionomycin were discarded.

The cells were stimulated with 100 µM histamine or 15 µM 2,5-Di-*t*-butyl-1,4-benzohydroquinone (BHQ: selective SERCA blocker) in the presence and absence of extracellular Ca^2+^, after incubation for 24 h with 25 µg/mL Poly I:C, 25 µg/mL dsRNA, 2.5 µg/mL Λ-DNA, 2.5 µg/mL total RNA or control solution.

### Live Cell Atomic Force Microscopy Measurements

The measurements were performed with an Asylum MFP-3D head and Molecular Force Probe controller (Asylum Research, Santa Barbara, CA, USA). The driver program MFP Xop was written in IGOR Pro software (version 5.0.3, Wavemetrics, Lake Oswego, OR, USA). The MFP-3D head was mounted on a Zeiss Axiovert 200 invert optical microscope. The cells were cultured on gelatine-coated 15 mm diameter glass cover slips until confluence. The sample was mounted on the stage of the microscope and contact mode image and force curve acquisition was performed. Afterwards the cells were treated with 25 µg/mL Poly I:C or left untreated (control). The image and force curve acquisition was repeated every 30 min for 3 hours. For imaging in solution, gold coated, silicon nitride, rectangular cantilevers were used (Bio-lever, BL-RC150 VB-C1, Olympus Optical Co. Ltd., Tokyo, Japan). The imaging in solution and the determination of the Young’s moduli were made as previously reported [37], [38].

### Cell Growth and Proliferation Assays

Cells were plated at a density of 10^4^ cells/well in 2%FBS/Medium in 96-well plates and were allowed to adhere overnight. The concentration- dependent effect of 24 h incubation with Poly I:C, dsRNA, totalRNA, Λ-DNA, LY-294002 or BHQ on growth and proliferation of hPAECs was determined by [^3^H]thymidine incorporation as an index of DNA synthesis. [^3^H]thymidine (0.2 µCi/well) was added for the last 16 h. After 24 h incubation the cells were harvested and transferred to filter-plates. The radioactivity was measured using a beta-counter.

### Flow Cytometry

To measure the cell-cycle dependent amount of DNA per cell, propidium-iodide staining was performed on methanol-fixed hPAECs after 24 h incubation with Poly I:C or control solution. The cells were trypsinized, centrifuged for 5 min at 220×g at 4°C, and then washed with 10 ml PBS. The pellet was re-suspended and again centrifuged for 5 min at 220×g at 4°C and dissolved in 500 µl PBS. For 10^6^ cells 5 mL of 70% ice cold methanol was added for 10 min at 4°C. For data analysis the *FCA3.3 DNA subG1* protocol was used.

### Transfection of Small Interfering RNA against SERCA3

Small interfering RNAs (siRNA) against SERCA3 were commercially synthesized (siSERCA3, sc-41295, Santa Cruz Biotechnology). As negative control, non-silencing RNA (siCTL, Control siRNA-A, sc-37007, Santa Cruz Biotechnology) which does not target any human gene product was used. Either 200 nM siCTL or 200 nM siSERCA3 was mixed with 500.000 hPAECs and 100 µl of Basic Nucleofector Solution from Basic Endothelial Cells Nucleofector Kit (Lonza). This mixture was electroporated according to the manufacturers’ instruction. Knock-down was confirmed by quantitative RT-PCR and western blot with specific primers and antibody. Permeability measurement with fluorescently labelled dextran and VE-cadherin staining were performed 48–56 h post transfection.

### Statistical Analysis

Numerical values are given as means ± SD of n cells. Intergroup differences were assessed by factorial analysis of variance with post hoc Fisher’s least significant difference test or Student’s t-test (p values <0.05 were considered significant).

For the live cell Ca^2+^ imaging data analysis, the basal level of Ca^2+^ was determined as an average value of the first 50 seconds of the curve. Afterwards, the histamine-induced Ca^2+^ peak height after subtracting the baseline as well as the plateau duration of the Ca^2+^ response were quantified. The plateau duration is the time from the maximum Ca^2+^ peak height until the intracellular Ca^2+^ declines to the basal level.

To determine the blocking potency of Poly I:C on hPAECs proliferation, concentration–inhibition curves were constructed from the FCS-induced hPAECs proliferation in the presence of different drug concentrations in the media. The proliferation values are given in the presence of Poly I:C as a fraction of the FCS-induced proliferation in the absence of Poly I:C. The normalised values were fitted by means of a nonlinear least-squares method with the equation: 1(1+c(IC50)–1)h)–1, where c was the drug concentration, IC50 was the concentration giving a half-maximum effect and h was the Hill coefficient. Because the Hill coefficient was less than 1, it was set to one, accounting for a 1∶1 binding stoichiometry.

## Results

### Modulation of the Human Pulmonary Artery Endothelial Monolayer Integrity by Double-stranded RNA

**Figure 1:**
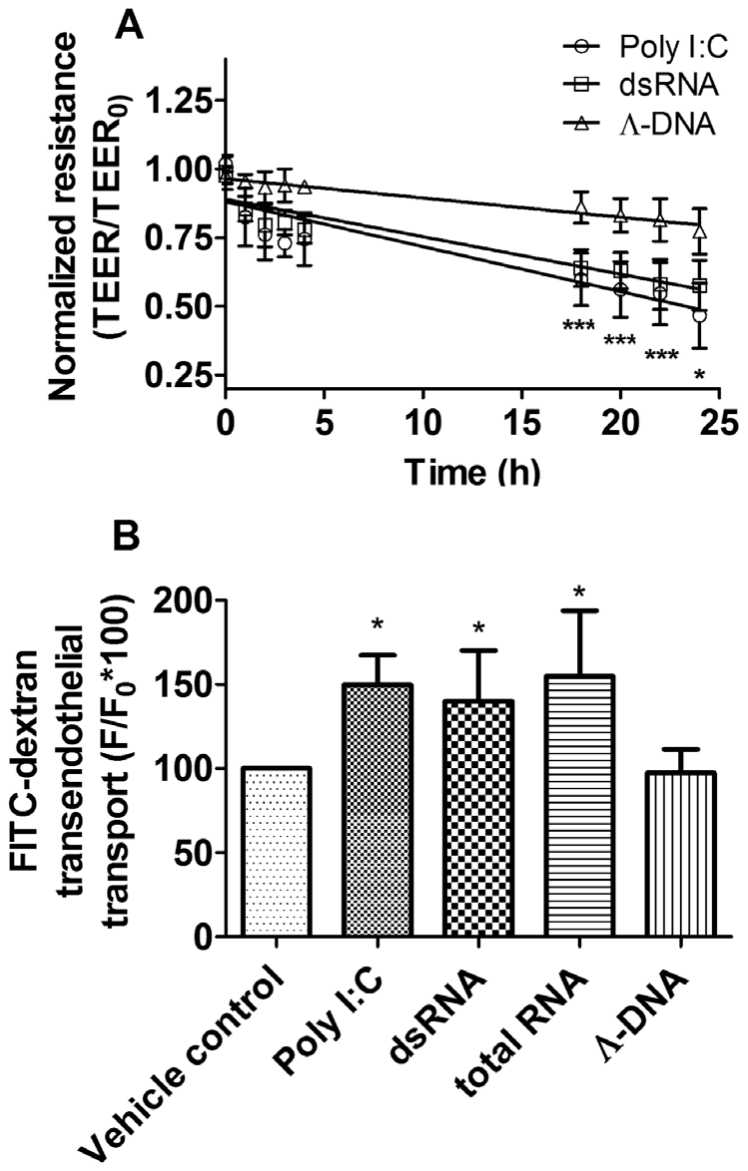
Double-stranded RNA decreased transendothelial electric resistance and increased FITC-dextran permeability through hPAECs monolayers. (A) Both natural double-stranded RNA (squares) and synthetic analogue (Poly I:C, circles) caused a significant, time dependent, linear decrease of the transendothelial electric resistance (TEER) of the human pulmonary artery endothelial cell (hPAEC) monolayer. Λ-DNA served as a control and had no significant effect. The graph summarizes the results of 6 independent measurements for each treatment and the linear fit of the data. The values were normalized to the TEER of hPAECs at the start of the experiment (*p<0.05, ***p<0.001 compared to Vehicle control). (B) Histogram summarizing the effect of the 24 h treatments on the FITC-dextran permeability of hPAEC. Double-stranded RNA significantly increased FITC-dextran traffic. Similar results were obtained after Poly I:C and total RNA treatment, but not with Λ-DNA. Values represent 3 independent experiments, each performed in triplicates and they were normalized to the vehicle control (*p<0.05 compared to Vehicle control).

Double-stranded RNA (dsRNA) or its synthetic analogue (Poly I:C) significantly decreased the electric resistance and increased the permeability of the confluent human pulmonary artery endothelial (hPAEC) monolayer as shown on Figure 1. Primary hPAECs were incubated with dsRNA or Poly I:C up to 24 hours and the transendothelial electric resistance (TEER) was measured (Figure 1A). Λ-DNA, which is not a toll-like receptor 3 ligand [8], was used as a control. Significant decrease in TEER was observed already after 18 hours of treatment suggesting that both, dsRNA and Poly I:C disrupted the integrity of the endothelial monolayer. In contrast, the incubation with Λ-DNA had no significant effect. Consistently, the trafficking of FITC-labeled dextran molecules through the confluent endothelial monolayer was significantly increased after 24 h dsRNA, Poly I:C or total RNA application (Figure 1B, 131±30%, 140±23% and 152±45% of control, respectively). 24 h incubation with Λ-DNA had no significant effect (98±15% of control).

**Figure 2:**
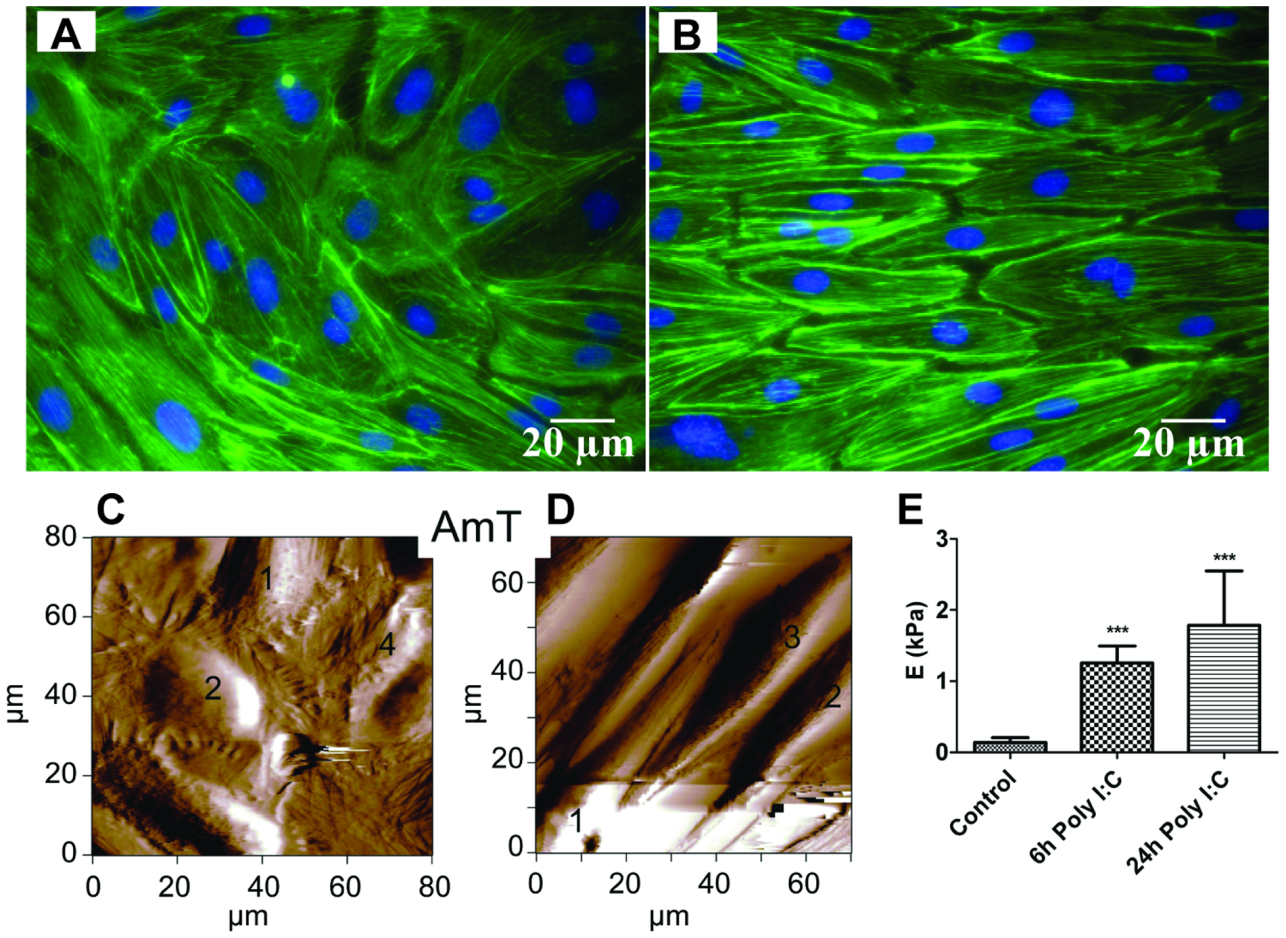
Actin rearrangement and stiffening of hPAECs under stimulation with Poly I:C. Actin rearrangement (green) showing changes in the cell shape (A - control, B - 24 h after Poly I:C). Nuclei were counterstained with DAPI (blue). (C) Representative 80×80 µm^2^ amplitude atomic force microscope image of hPAEC culture without (Control) and with 24 h Poly I:C treatment (D). (E) The force measurements (performed on 5 different points in the central region of numbered cells) showed a significant increase in the Young’s moduli of the cells (***p<0.001 compared to Control).

An elongation of the Poly I:C treated cells and the appearance of rearranged fiber structures was observed on the actin staining (Figure 2A, B) and more prominently in the live cell atomic force microscopy images (Figure 2C, D). The representative amplitude image of the control hPAECs showed a regular cell shape as seen in Figure 2C, whereas elongated cells were detected on the images taken from the 24 h Poly I:C treated culture (Figure 2D). The Poly I:C treatment was also associated with a time dependent stiffening of the cells (Figure 2E). The Young’s moduli of the hPAECs was determined by direct force measurements on 5 distinct points above the nuclear region of the numbered cells shown in Figure 2C and D. A significant increase in the Young’s moduli from 0.25±0.15 kPa to 2.15±1.5 kPa upon 24 h Poly I:C treatment was observed (Figure 2E).

**Figure 3:**
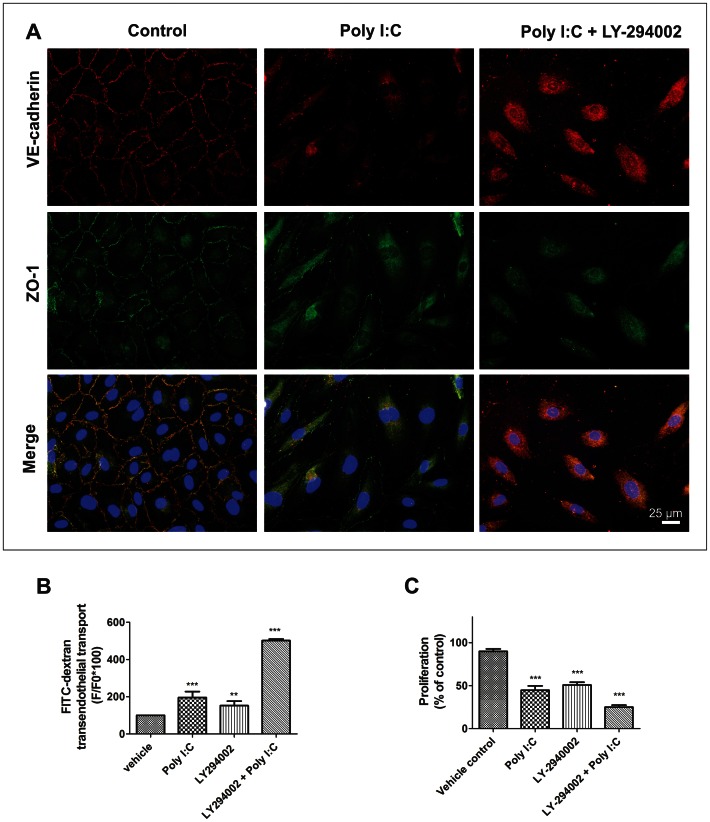
Involvement of PI3 kinase in the Poly I:C induced cell-cell contact disruption and permeability increase. (A) 24 h LY-294002 (PI3 kinase blocker) treatment along with Poly I:C (third column) reduced the VE-cadherin and ZO-1 signal similarly to 24 h Poly I:C treatment (second column) compared to control (first column). (B) LY-294002 significantly increased the FITC-dextran permeability of hPAEC, comparably to Poly I:C effect. The bar graph summarizes 3 independent experiments each performed in triplicates. (C). 24 h LY-294002 treatment significantly reduced hPAEC proliferation compared to Vehicle control. A similar and additive effect of 24 h Poly I:C treatment has been observed (**p<0.01, ***p<0.001 compared to Vehicle control).

The integrity of the intercellular junctions was further investigated by vascular endothelial-cadherin (VE-cadherin) and Zonula occludens 1 (ZO-1) staining. The continuous membrane staining for both VE-cadherin and ZO-1 showed colocalization of these two proteins (Figure 3A). 24 h of Poly I:C treatment caused disruption of the endothelial barrier integrity. The VE-cadherin and ZO-1 membrane staining either disappeared or became discontinuous (Figure 3B).

### Involvement of Phosphatidylinositol 3-Kinase in the Poly I:C induced Endothelial Dysfunction

Next, we investigated the TLR3 - Phosphatidylinositol 3-Kinase **(**PI3-Kinase) driven regulation of endothelial permeability and proliferation. Poly I:C treatment resulted in a 35-fold upregulation of TLR-3 in hPAECs (Figure S1, Methods S1). However, both VE-cadherin and ZO-1 stainings (Figure 3A) showed a cumulative effect of Poly I:C and LY-294002 (PI3-Kinase blocker) with a total disappearance of both proteins from the cell membranes. Consistently, the FITC-labelled dextran traffic was significantly increased by 24 hours of LY-294002 (152.10±24.18%) and this effect was additive with the Poly I:C effect, leading to a 501.80±8.27% increase in the amount of transmitted fluorescent dextran molecules (Figure 3B). Additionally, the proliferation of the hPAECs was blocked to 50.73±10.32% by LY-294002, similar to the Poly I:C effect (44.74±15.92%) and further inhibited to 24.95±7.44% when both compounds were administered together (Figure 3C).

### Effect of dsRNA on the Cell Cycle of hPAECs

**Figure 4:**
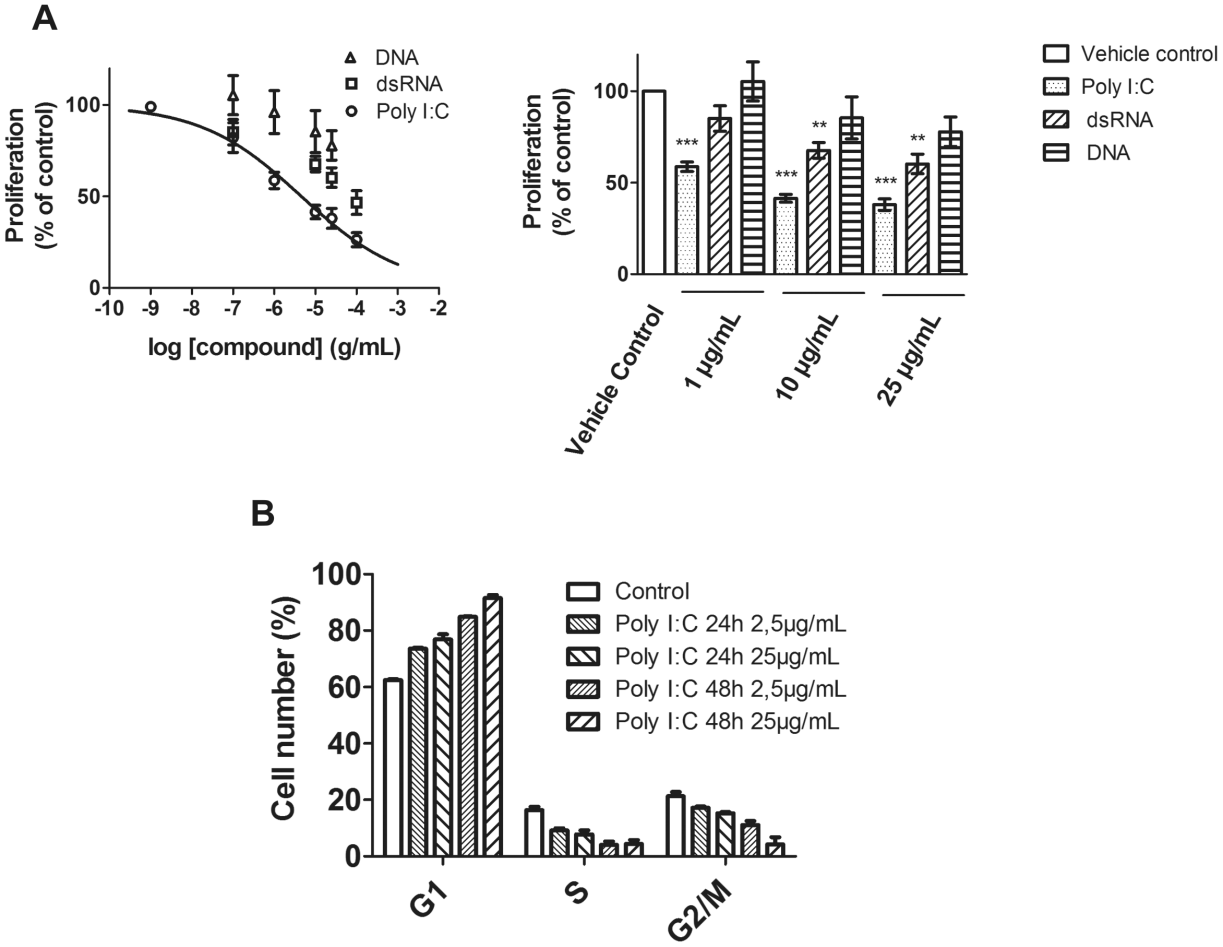
Proliferation inhibition of hPAEC by natural and synthetic dsRNA. (A) Concentration dependent inhibition of the hPAEC proliferation upon Poly I:C administration. The line is the best fit to the Hill equation with an IC_50_ of 2.0±0.3 µg/mL. The bar graph summarizes the effect on hPAEC proliferation of Poly I:C, double-stranded RNA and Λ-DNA treatment. The graphs represent 3 independent experiments, each performed in triplicates (**p<0.01, ***p<0.001 compared to Vehicle control). (B) Histogram summarizing the effect of Poly I:C treatment on the cell number distribution in G1, S and G2/M phase of cell cycle. The graph represents 3 independent experiments of flow-cytometric analysis of propidium iodide stained cells.

The 24 h Poly I:C or dsRNA treatment reduced dose-dependently the FCS-induced hPAECs proliferation (Figure 4A), whereas Λ-DNA had no significant effect. The concentration-response curve of the Poly I:C-induced inhibition of proliferation resulted in an IC_50_ of 2.0±0.3 µg/mL (Figure 4A; n = 5 for each group).

Furthermore, upon Poly I:C treatment a dose- and time- dependent accumulation of hPAECs in the G1-phase was observed (Figure 4B). The number of cells in the G1 phase was significantly increased from 62.4±0.3% (control) to 76.9±1.8% after 24 h and further raised to 91.5±1.1% after 48 h of treatment with 25 µg/mL Poly I:C. This increase was accompanied by a decrease in the number of cells both, in the S and in the G2/M phase.

### Impact of dsRNA on Intracellular Ca^2+^ Homeostasis and SERCA Activity of hPAECs

**Figure 5:**
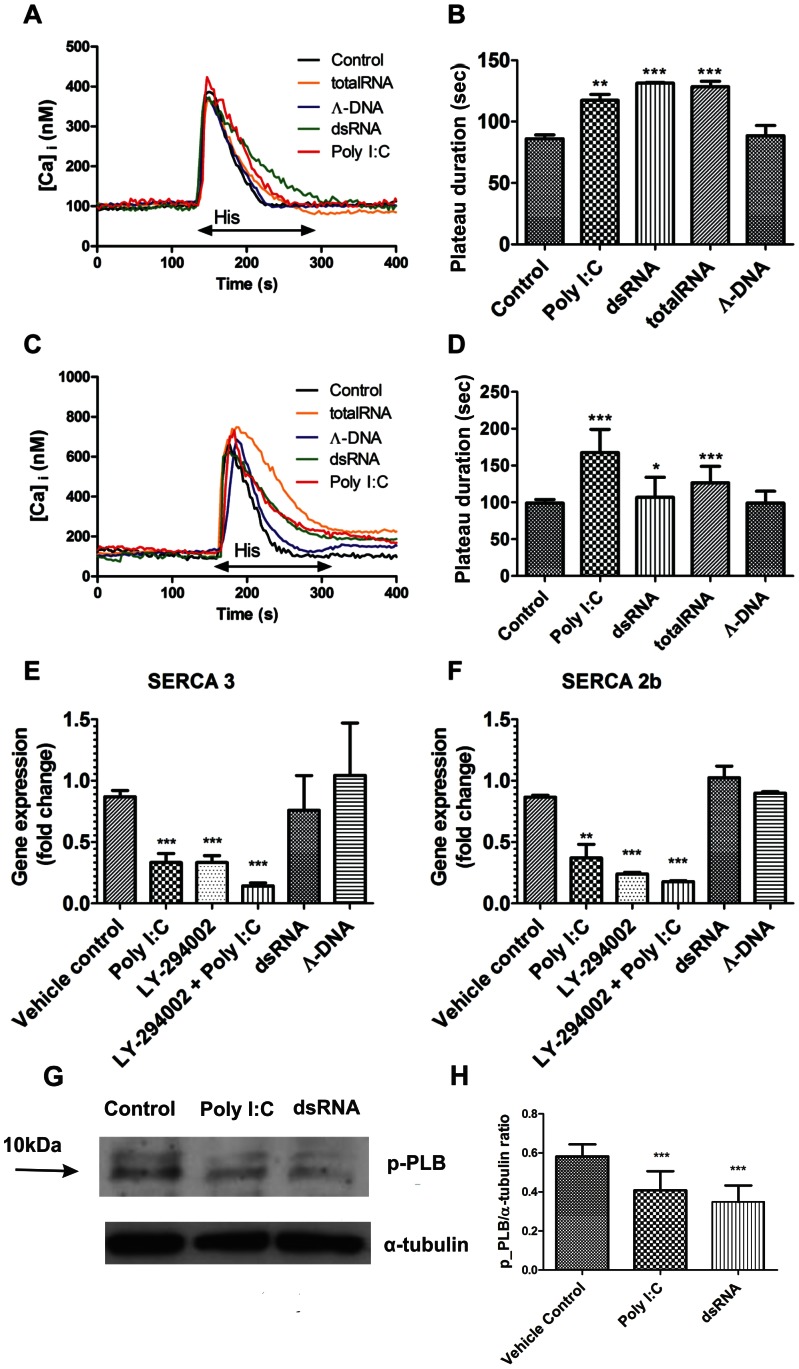
Prolonged histamine-induced Ca^2+^ plateau in hPAECs after Poly I:C incubation accompanied by SERCA downregulation and phospholamban dephosphorylation. Representative traces of 100 µM histamine induced intracellular Ca^2+^ rise under Ca^2+^-free (A) and Ca^2+^ (C) conditions showed a prolonged decay of intracellular Ca^2+^ level in hPAECs after 24 h Poly I:C (red line), dsRNA (green line) or total RNA (orange line) treatment. As a control, Λ-DNA had no effect (blue line). Arrow indicates the application of histamine (His). (B) The histamine induced transient Ca^2+^ plateau duration was significantly longer in the case of treatment (*p<0.05, **p<0.01, ***p<0.001 compared to Control, untreated sample) in the absence (B) and presence (D) of 1.8 mM extracellular Ca^2+^. Bar graphs show expression of SERCA3 (E) and SERCA2b (F) isoform of the sarco-endoplasmic reticulum Ca^2+^ ATPase pump. Results are from 3 independent experiments each performed in triplicates (**p<0.01, ***p<0.001 compared to Vehicle control). (G) Phospholamban phosphorylation upon 24 h Poly I:C and dsRNA treatment (p-PLB - phosphorylated phospholamban). (H) Bar graph represents p-PLB/α-tubulin ratio from 3 independent western blot experiments (***p<0.001 compared to Vehicle control).

Because changes in intracellular Ca^2+^ concentration affect cell proliferation, the role of dsRNA on [Ca^2+^]_i_ was further analyzed in hPAECs. Independently of the extracellular Ca^2+^ concentration, there was no significant effect on the resting Ca^2+^ concentration (Table 1) and on the 100 µM histamine-induced peak Ca^2+^ concentration of the cells (Table 2). In contrast, 24 h dsRNA incubation significantly prolonged the 100 µM histamine-induced Ca^2+^ signal in the absence (Figure 5A) or presence of extracellular calcium (Figure 5C). Similar results were observed with Poly I:C, whereas Λ-DNA incubation had no effect (Figure 5). This prolonged Ca^2+^ signal (Figure 5B, D) suggested that dsRNA treatment caused a delay in the clearance of cytoplasmic Ca^2+^, which is necessary for replication.

**Table 1:** Basal Ca^2+^ concentration of hPAECsin the presence and absence of extracellular Ca^2+^.

Protocol	Baseline(nM Ca)	SD	Nr. of cells
**Ca^2+^ control**	128.21	75.89	41
**Ca^2+^-free control**	134.13	90.84	59
**Ca^2+^-free Poly I:C**	108.43	77.90	44
**Ca^2+^ Poly I:C**	110.72	110.92	29

**Table 2:** Histamine induced Ca^2+^ peak height of hPAECs in the presence and absence of extracellular Ca^2+^.

Protocol	Peak height(nM Ca)	SD	Nr. of cells
**Ca^2+^ control**	364.16	162.99	33
**Ca^2+^-free control**	383.24	299.02	41
**Ca^2+^-free Poly I:C**	326.14	250.09	24
**Ca^2+^ Poly I:C**	312.74	127.09	22

To further investigate the effect of dsRNA on the sarco-endoplasmic reticulum Ca-ATPase (SERCA), gene expression analysis was performed. Both SERCA isoforms (SERCA2b and 3) present in hPAECs showed a significant decrease in mRNA expression after 24 h of Poly I:C treatment (Figure 5E, F; 0.37±0.19 fold change for SERCA 2b and 0.33±0.13 for SERCA3). Treatment with the PI3 kinase blocker (LY-294002) caused a similar decrease in the SERCA expression (0.24±0.03 for SERCA 2b and 0.33±0.09 for SERCA3). Furthermore, the combination of LY-294002 and Poly I:C showed a cumulative effect (Figure 5E, F, 0.18±0.01 for SERCA 2b and 0.14±0.04 for SERCA3). In contrast, Λ-DNA did not alter the expression levels of these genes (0.90±0.03 for SERCA 2b and 1.04±0.73 for SERCA3).

Next, the effect of Poly I:C and dsRNA treatment on the phosphorylation of phospholamban, an endogenous inhibitor of SERCA was assessed. After 24 h treatment a significant decrease in the phospholamban phosphorylation was observed (Figure 5G, H).

**Figure 6:**
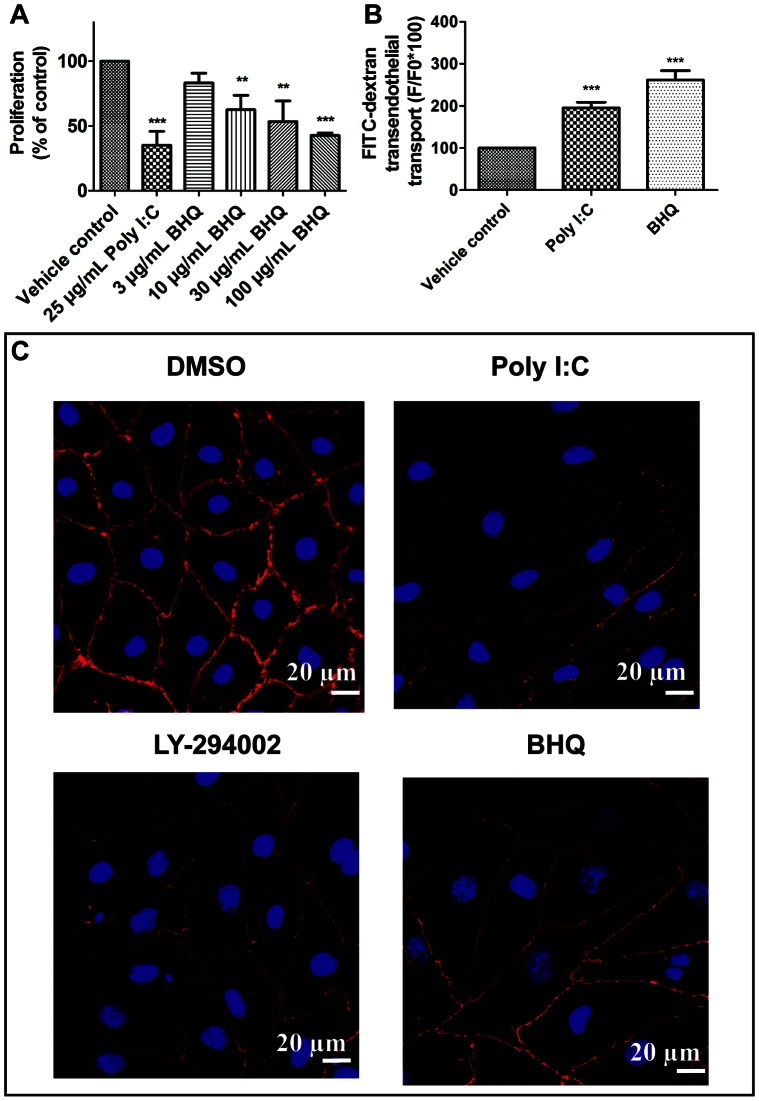
Inhibition of hPAEC proliferation, increase of FITC-dextran permeability and disruption of intercellular junctions by the SERCA blocker. (A) The SERCA blocker, BHQ inhibited hPAECs proliferation in a concentration-dependent manner. The bar graph summarizes 3 independent experiments each performed in triplicates. (B) BHQ significantly increased the FITC-dextran permeability of hPAECs. The bar graphs summarize 3 independent experiments each performed in triplicates. (**p<0.01, ***p<0.001 compared to Vehicle control). (C) Confocal microscopic images revealed that BHQ reduced the VE-cadherin signal compared to control, similar to Poly I:C and LY-294002. Nuclei were counterstained with DAPI (blue).

**Figure 7:**
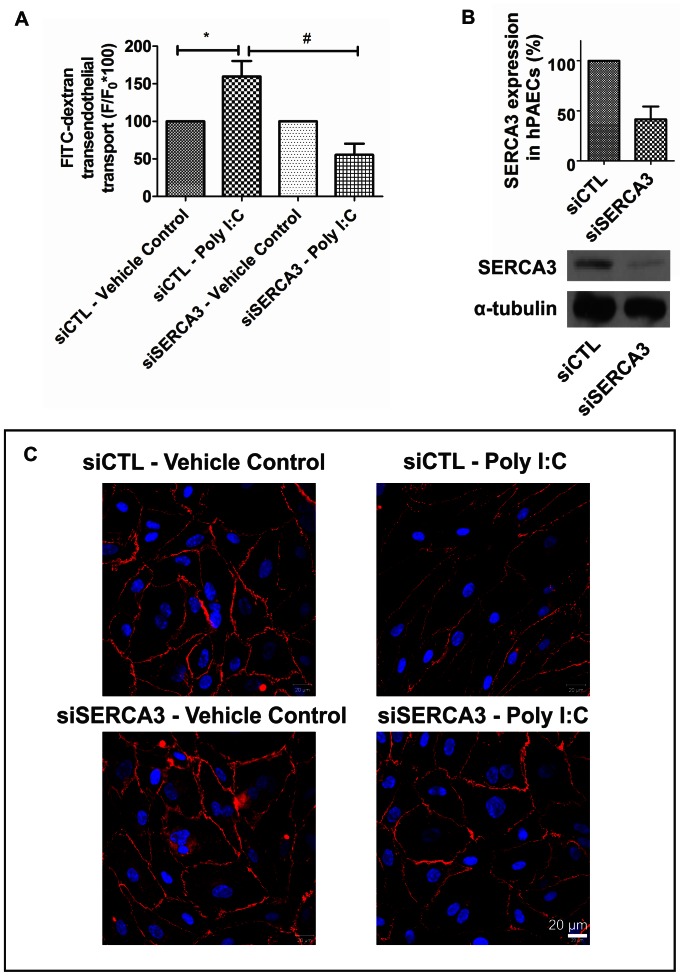
siRNA treatment against SERCA3 protects the hPAECs from Poly I:C induced permeability and junctional changes. (A) siRNA silencing of SERCA3 abolished the FITC-dextran permeability increase of hPAECs caused by Poly I:C. The bar graphs summarize 4 independent experiments each performed in triplicates. (*p<0.05 compared to siCTL - Vehicle control, ^#^p<0.001 compared to siCTL - Poly I:C). (B) mRNA and protein level of SERCA3 upon treatment of siCTL and siSERCA3. (C) Representative confocal microscopic images reveal that siSERCA3 treated hPAECs respond with less VE-cadherin signal loss compared to siCTL treated hPAECs upon 24 h Poly I:C stimulation. Nuclei were counterstained with DAPI (blue).

While an inhibitory effect of dsRNA on the SERCA pump was expected, we investigated the SERCA blocker (BHQ) induced dose-dependent inhibition of the hPAEC proliferation (Figure 6A). The effect was similar to that of dsRNA or Poly I:C (Figure 4A). BHQ treatment led to disruption of the VE-cadherin staining similar to Poly I:C or LY-294002 (Figure 6C). This effect was accompanied by increase in the endothelial permeability (Figure 6B) pointing to decreased endothelial barrier function. Next, we investigated the effect of Poly I:C after siRNA silencing of SERCA3 on hPAECs (Figure 7). The observed increase in permeability upon 25 µg/mL Poly I:C treatment on siCTL hPAECs, was significantly reduced with the silencing of SERCA3 (Figure 7A). Parallel, confocal microscopic images revealed that knock down of SERCA3 caused less VE-cadherin signal loss upon 24 h Poly I:C stimulation as compared to siCTL (Figure 7C). The silencing of SERCA3 confirmed the previous findings obtained with the SERCA blocker, BHQ.

Altogether, these data suggest that synthetic dsRNA treatment alters the function of SERCA, which inhibits cell proliferation by inducing G1 arrest in the hPAECs and contributing to endothelial dysfunction.

## Discussion

The major function of the vessel-forming endothelial cells is to maintain the blood-tissue barrier. Viral RNA has been identified as a pathogenic factor in different pulmonary diseases [39], [40]. Circulating RNA may lead to pulmonary endothelial dysfunction and thus to increased endothelial permeability [8]. The precise mechanism by which circulating RNA induces endothelial barrier disruption in the lung has not been completely understood yet. The present study addressed novel pathways of dsRNA effect on primary human pulmonary artery endothelial cells (hPAECs). Our data showed that natural dsRNA treatment significantly decreased electric resistance indicating disruption in the hPAEC monolayer integrity. A similar effect could be observed with the widely used synthetic dsRNA-analog, Poly I:C. Incubation with Λ-DNA did not cause significant permeability changes indicating the specificity of the dsRNA effect. As dsRNA and Poly I:C treatment mimics viral infection, our results are in line with previous observations on bluetongue virus-infected human primary microvascular endothelial cells [41]. The EC monolayer disrupting effect of dsRNA was further confirmed by using fluorescently labeled dextran trafficking, showing that stimulation with either Poly I:C, total RNA or dsRNA resulted in an increase in transferred dextran amount suggesting a significant loss in endothelial barrier function.

The tight, isolating monolayer of the endothelial cells is accomplished by junctional structures, like adherens and tight junctions. The major component for the maintenance of monolayer integrity belongs to the transmembrane protein family, the cadherins [21]. The vascular endothelial cells express in their membrane the vascular endothelial cadherin (VE-cadherin) member of this family. Poly I:C incubation decreased VE-cadherin signal, pointing to disrupted cell-cell contacts in the monolayer. A similar decrease was reported as a result of dengue-2 virus infection in human umbilical vein endothelial cells [21]. We observed a similar decrease in the ZO-1 protein upon Poly I:C treatment. Furthermore, our results indicate that not only the junctional proteins contribute to dissociation of intercellular contacts, but also the actin reorganization. The Poly I:C treated cells presented with an intense peripheral actin staining accompanied by cellular shape changes visible on the immunofluorescent pictures. Similar cytoskeletal rearrangement was shown to be induced by 6 h bluetongue virus infection in primary human microvascular endothelial cells [41]. Although, the authors report on a recovery of both electrical resistance and actin/VE-cadherin staining after 24 h, this was not the case in either dsRNA, or Poly I:C treatment in our experiments. This could be explained by the differences between the human lung microvascular and arterial endothelial cells.

To investigate the possible involvement of the PI3-kinase pathway in the Poly I:C induced permeability and structural changes, we tested the effect of a PI3-Kinase blocker (LY-294002) on permeability, proliferation and junctional staining. It has been shown that the TLR3 - PI3-Kinase pathway could be involved in the dsRNA induced signalling [42]. Indeed, we observed an upregulation of the TLR3 receptor upon Poly I:C treatment. The block of PI3-Kinase resulted in increased hPAEC permeability, decreased proliferation and disruption of VE-cadherin and ZO-1 staining. On primary hPAECs, the blocking was additive to the effect of Poly I:C, pointing to a PI3-Kinase independent mechanism of action. However, others report that PI3-Kinase is involved in dsRNA signalling [42]. In their study, a mammalian heterologuos expression system (HEK293) was used with a higher concentration of Poly I:C. We could confirm the inhibitory effect of PI3-kinase on SERCA expression, as it was previously reported with a PI3-kinase inhibitor on SERCA dependent calcium handling [43]. However, the effect on downregulation of both isoforms was additive pointing to a PI3-Kinase independent mechanism of action, similar to the effects we observed on hPAECs permeability and proliferation.

Structural changes caused by inflammatory stimuli may lead to dissociation of cell-cell junctions leading to a widened intercellular space that facilitates transendothelial flux [1]. We observed similar structural rearrangement in light microscopy: the Poly I:C treated cells presented a morphological change with an elongated shape. *In-situ* atomic force microscopy (AFM) imaging and force measurements detected structural changes in the endothelial cells at single cell level. Endothelial barrier-disruptive agents have been reported to cause changes in cytoskeletal mechanics of hPAECs [44]. In our AFM images, cells treated with Poly I:C showed an elongated morphological shape. Furthermore, the Poly I:C treatment also led to an approximately 10-fold stiffening of the cells. This is in line with the observations of the effect of other barrier-disruptive agents on hPAECs [44], [45]. The change in the mechanical properties of the cells can lead to disturbed or disrupted cell-cell contacts and may contribute to endothelial dysfunction.

A critical factor for the regular endothelial function is the Ca^2+^ homeostasis [25]. Our data suggest that, beside structural changes, Poly I:C and dsRNA also affects endothelial Ca^2+^ homeostasis. Acute administration of either Poly I:C, or dsRNA did not result in any Ca^2+^ response, but 24 hours of treatment led to a significantly prolonged histamine-induced Ca^2+^ response, without affecting the basal Ca^2+^ level. Further, we focused on intracellular Ca^2+^ mobilization. The key player of the Ca^2+^ store refilling is the sarco-endoplasmic reticulum Ca-ATPase (SERCA) [28] and its proper regulation is vital for normal cell function [33]. An inhibitory effect of Poly I:C on SERCA is suggested by the increased duration of the histamine-induced signal, observed in the presence and absence of extracellullar Ca^2+^. We observed a decrease in the phospholamban phosphorylation upon Poly I:C or dsRNA treatment, which contributes to enhanced block of SERCA function. Phospholamban is a negative regulator of SERCA function [46]. Phospholamban phosphorylation causes its dissociation from SERCA and removes inhibition [47].

RT-PCR showed a decrease in the expression of both isoenzymes of SERCA upon Poly I:C treatment. However, upon dsRNA treatment no changes in the gene expression could be observed. Thus, our data indicate, that dsRNA does not regulate SERCA expression like its synthetic analogue (Poly I:C). Nonetheless, both caused decrease in the phospholamban phosphorylation. Furthermore, the SERCA blocker (BHQ) induced a dose-dependent inhibition of the hPAEC proliferation, a decrease in the VE-cadherin staining and increase in the transendothelial transport similar to the effect of dsRNA or Poly I:C. The siRNA silencing of SERCA3 on hPAECs confirmed the involvement of SERCA in the Poly I:C induced endothelial dysfunction.

Our data indicate that Poly I:C induces G1 arrest in the hPAECs by inhibiting the function of SERCA, which is vital for cell cycle control. In rat aortic endothelial cells, a decrease in SERCA activity resulted in a delayed G1 to S phase transition in the cell cycle [33]. SERCA dysfunction has been also reported following exposure of pancreatic beta cells to cytokines [48]. However, in our experiments not only the function, but also the expression of SERCA decreased upon Poly I:C treatment, which could result in disturbance of cell cycle regulation [33]. Exposure of hPAECs to Poly I:C in fact disturbed the regular cell cycle. Furthermore, a dose and time-dependent accumulation of hPAECs in the G1-phase was observed. This increase was accompanied by decrease in the number of cells both, in S and in G2/M phase, indicating that the cell cycle is arrested. In addition, Poly I:C treatment resulted in inhibition of proliferation, with no increase in apoptosis (Figure S2, Methods S1). As the Ca^2+^ homeostasis of the cells was altered through affecting the SERCA pump but without change in apoptosis, we conclude that Poly I:C causes Ca^2+^ mishandling, due to SERCA downregulation and functional inhibition, thereby leading to G1 arrest and finally to inhibition of hPAEC proliferation.

In conclusion, our data suggest that exposure to synthetic double-stranded RNA modulates Ca^2+^ signaling in human pulmonary artery endothelial cells by inhibiting their Ca^2+^-extruding pump, the sarco-endoplasmic Ca^2+^ ATPase. The cell cycle and the cell monolayer integrity are affected resulting in an accumulation of the hPAECs in G1 phase of the cell cycle. The circulating dsRNA due to reducing phospholamban phosphorylation may alter intracellular Ca^2+^ homeostasis and thus cell growth, leading to endothelial cell dysfunction. It is tempting to hypothesize that circulating extracellular RNA (e.g. in viral infection) acts as a barrier disrupting agent and contributes to the development of human pulmonary vascular dysfunction.

## Supporting Information

Figure S1 **Bar graphs represent the TLR3 gene fold change compared to untreated control after 24 hours of Poly I:C or Λ-DNA stimulation.**
(TIF)

Figure S2 Bar graphs represent percentage of apoptotic cells as measured by PI and Annexin V staining upon 24 hours of stimulation (Stauro - staurosporin; ***p<0.001 as compared to Control).
(TIF)

Methods S1 **Quantitative RT-PCR of TLR3 and apoptosis measurements in hPAECs.**
(DOCX)
