# Supplementary figures and images for: Double-Stranded RNA Attenuates the Barrier Function of Human Pulmonary Artery Endothelial Cells
Source: PLoS One. 2013 Jun 3;8(6):e63776. doi: 10.1371/journal.pone.0063776 (PMC3670875; doi:10.1371/journal.pone.0063776)

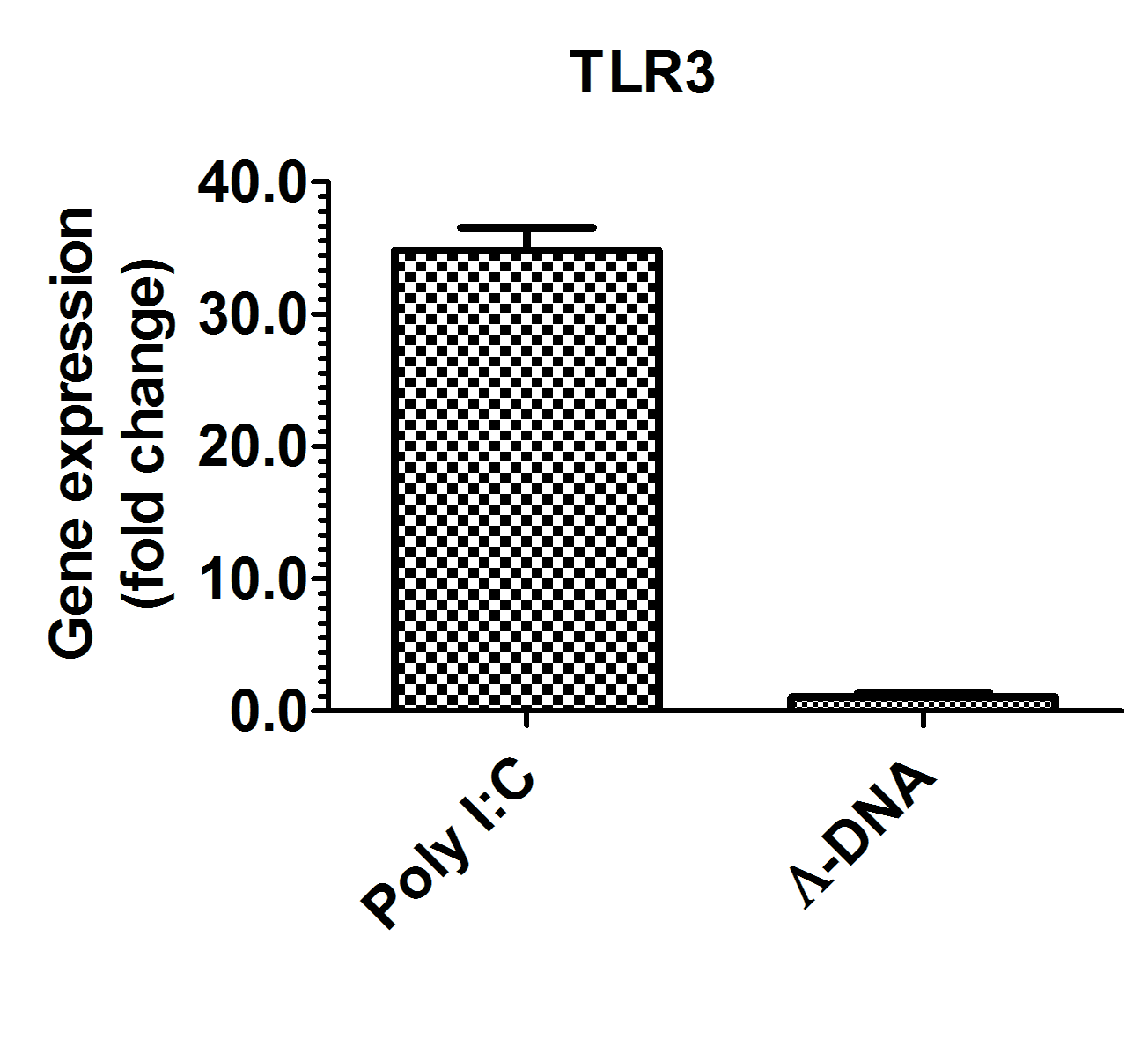

Supplement: Figure S1 — Bar graphs represent the TLR3 gene fold change compared to untreated control after 24 hours of Poly I:C or Λ-DNA stimulation. (TIF) [file pone.0063776.s001.tif]

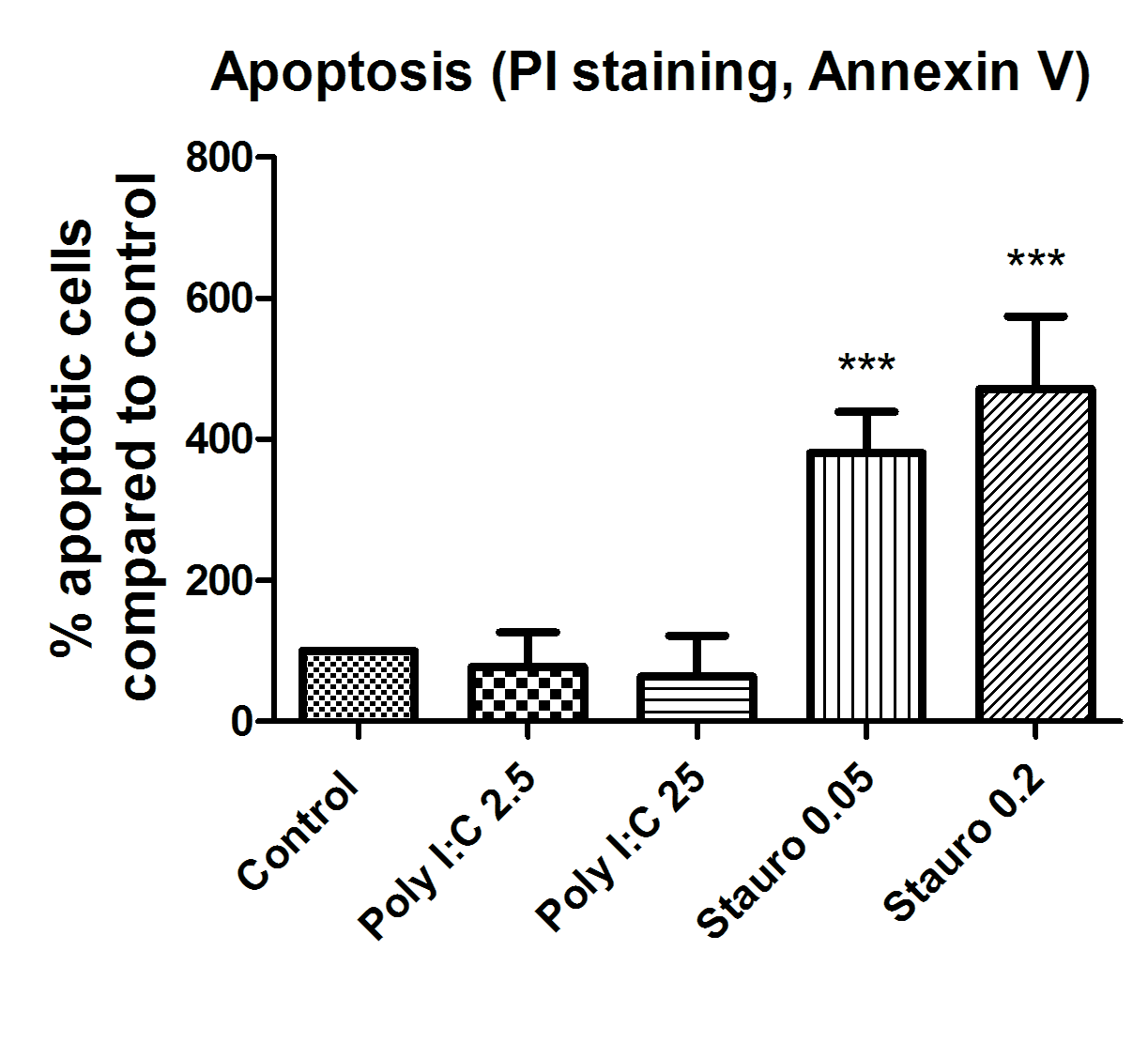

Supplement: Figure S2 — Bar graphs represent percentage of apoptotic cells as measured by PI and Annexin V staining upon 24 hours of stimulation (Stauro - staurosporin; ***p<0.001 as compared to Control). (TIF) [file pone.0063776.s002.tif]
