# Supplementary material for: Double-Stranded RNA Attenuates the Barrier Function of Human Pulmonary Artery Endothelial Cells
Source: PLoS One. 2013 Jun 3;8(6):e63776. doi: 10.1371/journal.pone.0063776 (PMC3670875; doi:10.1371/journal.pone.0063776)
Supplement: Methods S1 — Quantitative RT-PCR of TLR3 and apoptosis measurements in hPAECs. (DOCX) [file pone.0063776.s003.docx]

**Supporting Information (Methods S1)**

**Double-stranded RNA attenuates the barrier function of**

**human pulmonary artery endothelial cells**

Zoltán Bálint et al

**Supporting Materials and Methods**

1. Quantitative RT-PCR: HPAECs were incubated for 24h with 25 µg/ml Poly I:C, 2,5 µg/ml Λ-DNA or kept untreated. Total RNA was isolated from the cells and converted into cDNA using the RevertAid H Minus First Strand cDNA Synthesis kit (Fermentas). SYBR Green RT-PCR amplifications were performed by the AB7900 Syllabus using the following primer: Hs_TLR3_1_SG QuantiTect Primer Assay (200), nr. QT00007714 (Qiagen). Measurements were performed in triplets and the mean threshold cycle (Ct) reading was used. The target gene expression was quantified relative to the housekeeping gene (GAPDH) and normalized to the expression level of untreated control samples, which were set to 1.0 (delta-delta Ct method).

2. Apoptosis measurements: Cells were plated in culture medium in 25cm^2^ culture flasks and were allowed to adhere overnight. The cells were collected by centrifugation after trypsinization and stained with fluorescein-conjugated annexin V and propidium iodide (BD Biosciences, Germany). The degree of apoptosis after 24h incubation with 2.5 or 25µg/mL Poly I:C treatment of hPAECs was determined as the number of Annexin V positive cells by flow cytometry using a FACSCalibur (BD Biosciences, Germany). DMSO treated cells were used as vehicle control and 0.05 or 0.2µM Staurosporin (Cayman Europe) as a positive control. Cells that were propidium iodide-positive, Annexin V-negative were not counted as apoptotic cells.
